# The Golgi Localization of GOLPH2 (GP73/GOLM1) Is Determined by the Transmembrane and Cytoplamic Sequences

**DOI:** 10.1371/journal.pone.0028207

**Published:** 2011-11-29

**Authors:** Longbo Hu, Leike Li, Hongbin Xie, Yanli Gu, Tao Peng

**Affiliations:** 1 State Key Laboratory of Respiratory Diseases, Guangzhou Institute of Biomedicine and Health, Chinese Academy of Sciences, Guangzhou, China; 2 University of Science and Technology of China, Hefei, China; Institute of Molecular and Cell Biology, Singapore

## Abstract

Golgi phosphoprotein 2 (GOLPH2) is a resident Golgi type-II membrane protein upregulated in liver disease. Given that GOLPH2 traffics through endosomes and can be secreted into the circulation, it is a promising serum marker for liver diseases. The structure of GOLPH2 and the functions of its different protein domains are not known. In the current study, we investigated the structural determinants for Golgi localization using a panel of GOLPH2 truncation mutants. The Golgi localization of GOLPH2 was not affected by the deletion of the C-terminal part of the protein. A truncated mutant containing the N-terminal portion (the cytoplasmic tail and transmembrane domain (TMD)) localized to the Golgi. Sequential deletion analysis of the N-terminal indicated that the TMD with a positively charged residue in the cytoplasmic N-terminal tail were sufficient to support Golgi localization. We also showed that both endogenous and secreted GOLPH2 exist as a disulfide-bonded dimer, and the coiled-coil domain was sufficient for dimerization. This structural knowledge is important for the understanding the pathogenic role of GOLPH2 in liver diseases, and the development of GOLPH2-based hepatocellular cancer diagnostic methods.

## Introduction

Golgi phosphoprotein 2 (GOLPH2, also termed GP73 and GOLM1) is a type II transmembrane protein residing in the cis and medial-Golgi cisternae. GOLPH2 is predominantly expressed in the epithelial cells of many human tissues [1]. Abnormally increased expression of GOLPH2 has been reported to correlate with many diseases and viral infections. GOLPH2 overexpression has first been identified in acute giant-cell hepatitis, an uncommon form of hepatitis with a presumed viral etiology [1], and then in a variety of acute and chronic liver diseases [2], [3], [4], [5]. Fucosylated glycosylation has also been found in three quarters of secreted GOLPH2 from hepatocellular carcinoma patients [6]. Earlier studies have indicated that both the serum level of GOLPH2 and fucos-studded GOLPH2 could be a more reliable biomarkers for the early diagnosis of liver diseases than current markers like alpha-fetoprotein [7], [8].

Despite its potential as a biomarker for liver disease, knowledge on the structure and function of GOLPH2 remains very limited. Sequence analysis shows that GOLPH2 is highly conserved in vertebrates. It has a short N-terminal cytoplasmic domain followed by a transmembrane domain (TMD), and a longer C-terminal domain located in the Golgi lumen with a coiled-coil domain immediately beside the TMD [1]. To determine the possible physiological role of GOLPH2, Wright *et al.* constructed a transgenic mouse model with part of the GOLPH2 C-terminal truncated. GP73^tr/tr^ mice exhibited decreased survival and severe epithelial abnormalities in the liver and kidneys, suggesting that GOLPH2 may play an important role in epithelial cell function in these organs [9].

Being a type II transmembrane protein, GOLPH2 is unlikely to be a secreted protein. A possible explanation for its secretion comes from the observations that GOLPH2 is capable of intracellular trafficking between the Golgi and plasma membrane through an endosomal pathway, as well as the existence of a conserved proprotein protease cleavage site (R^52^VRR^55^) [10]. Cleavage by cellular proprotein convertase results in the secretion of the Golgi luminal portion of GOLPH2. The level of this proteolytic cleavage is speculated to be correlated with the level of GOLPH2 abnormal overexpression [10]. However, details on the structural determinants for GOLPH2 Golgi localization and intracellular trafficking are not clear.

The subcellular localization of Golgi proteins is signal dependent. Many type II membrane proteins with a short cytoplasmic tail contain a Golgi retention signal in or around their TMDs [11], [12], [13]. Two different models are proposed for Golgi localization. The oligomerization model posits that the formation of oligomers prevents protein movement into transport vesicles. In contrast, the bilayer-thickness model suggests that a short TMD restricts the protein to the thinner lipid bilayer of the Golgi, whereas a longer TMD allows the protein to be localized to the thicker lipid bilayer of the plasma membrane [14], [15].

To illustrate the structural determinants of GOLPH2 Golgi localization and its oligomeric status, a panel of GOLPH2 truncation mutants was constructed in the present study. Our data showed that the short N-terminal cytoplasmic tail and TMD were not only responsible for GOLPH2 intracellular trafficking and secretion, but were also required for the Golgi localization of GOLPH2. Our study also suggested that this Golgi localization depended on a positively charged residue at the cytoplasmic end, and on the length of the TMD. We found as well that both intracellular and secreted GOLPH2 exist as disulfide-bonded dimers mediated via the coiled-coil domain. Given that GOLPH2 is a promising marker for liver diseases, a detailed understanding of its function and structure could provide mechanistic insights into the pathogenesis of GOLPH2-related diseases.

## Results

### The N-terminal cytoplasmic tail and TMD are important for GOLPH2 trafficking

**Figure 1:**
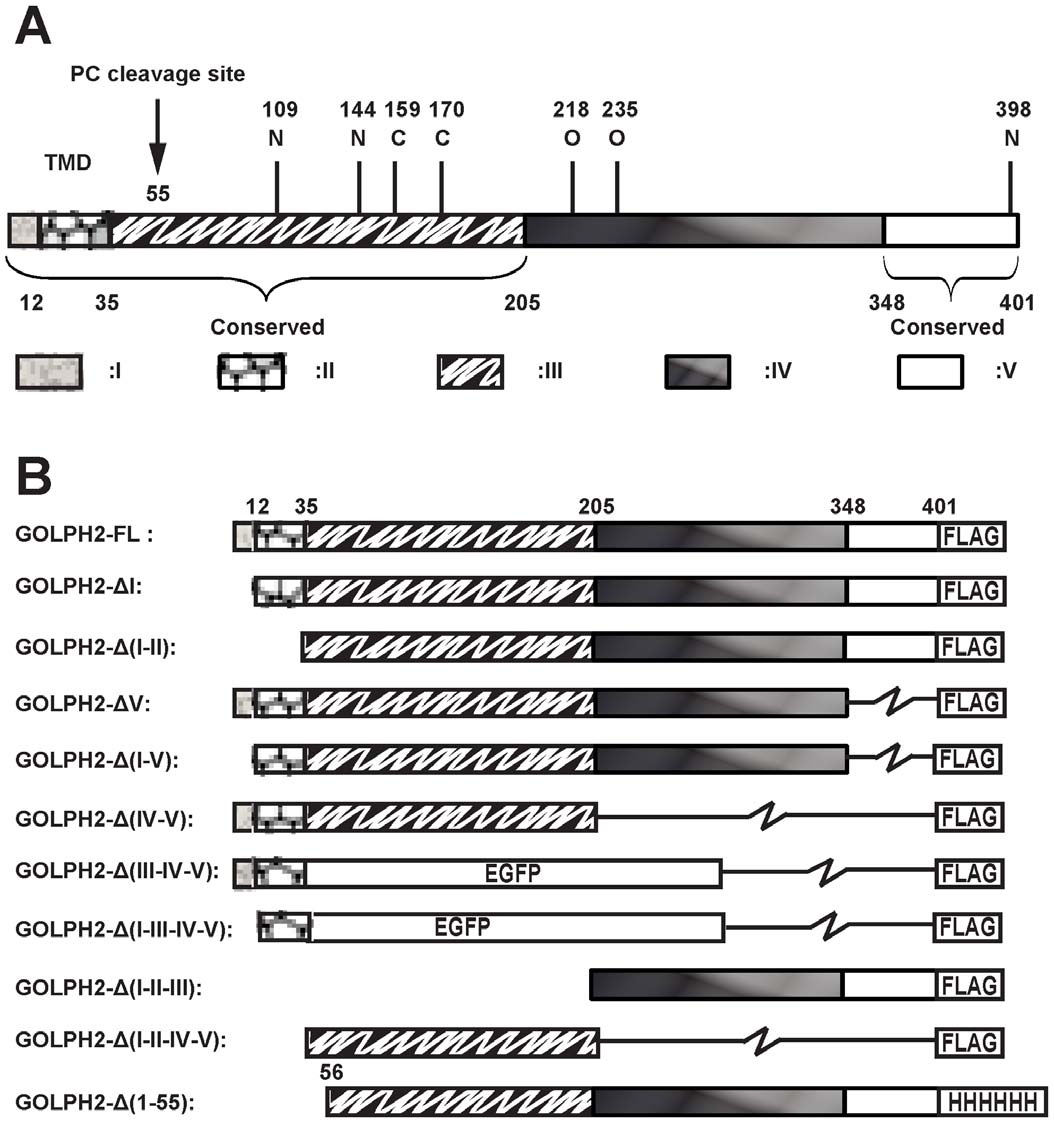
Schematic diagrams of GOLPH2 truncation mutants. **A. Structural regions of GOLPH2.** Based on sequence and structure analyses, GOLPH2 is divided into five regions: region I (cytoplasmic tail), region II (transmembrane domain), region III (coiled-coil domain), region IV, and region V (conserved domain of C-terminal). Five potential glycosylation sites are presented, namely, three N-linked glycosylation sites (N109, N144, and N398) and two O-linked glycosylation sites (T218 and T235). **B. GOLPH2 truncation mutants.** Different regions of GOLPH2 were amplified by PCR and cloned into pCR3.1 vector with an in-frame FLAG-tag encoded at the C-terminal to construct the GOLPH2 truncated mutants. A BamH I site was introduced into GOLPH2-Δ(IV–V) and GOLPH2-ΔI by point mutation. The EGFP coding region was cloned into GOLPH2-Δ(IV–V) and GOLPH2-ΔI in site with BamH I and EcoR I to generate GOLPH2-Δ(III–IV–V) and GOLPH2-Δ(I–III–IV–V) mutants.

**Figure 2:**
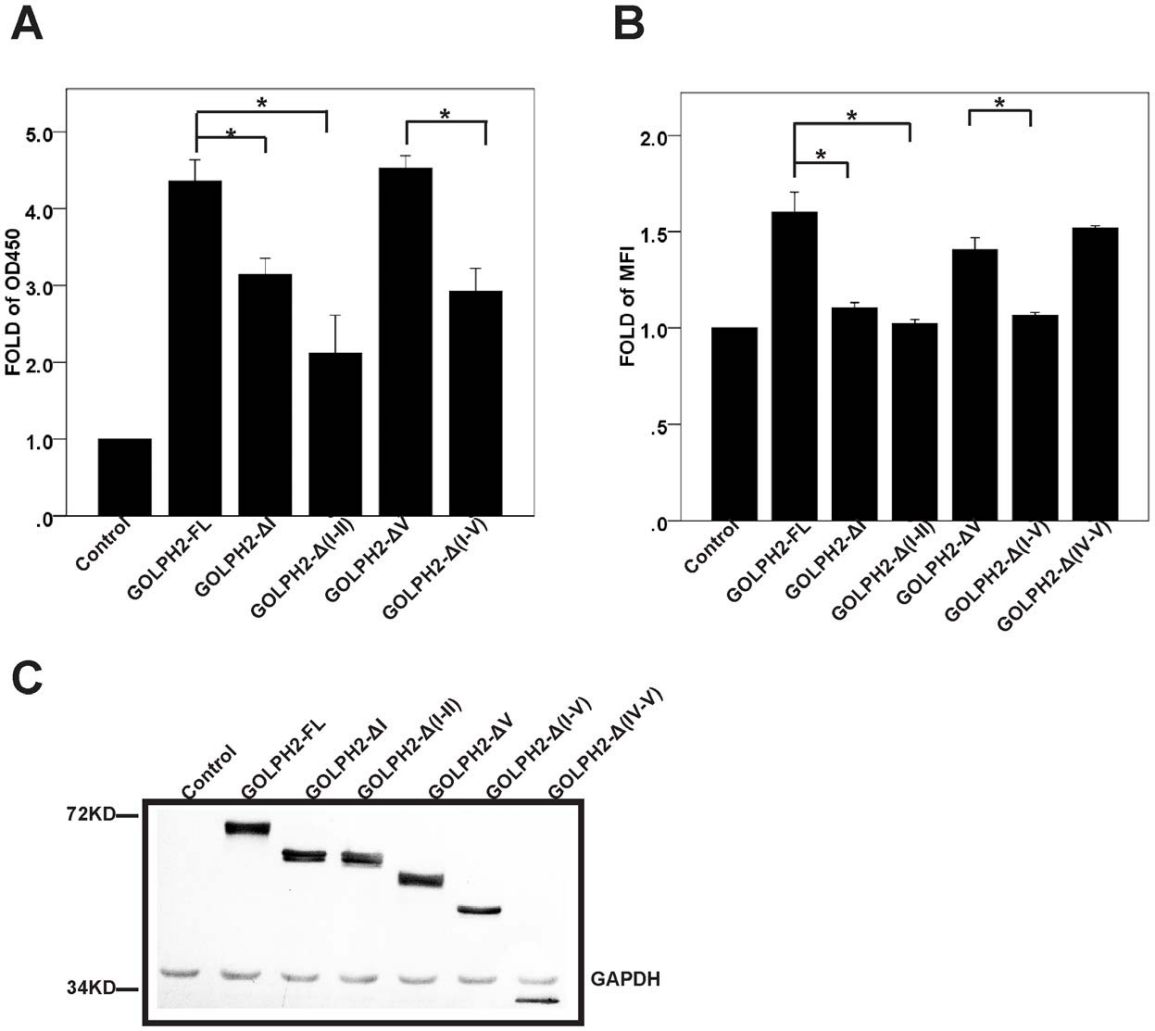
The TMD and cytoplasmic tail are important for GOLPH2 intracellular trafficking. **A. Secretion assay of GOLPH2 truncation proteins in HeLa cells.** HeLa cells were transfected with GOLPH2 truncation expression plasmids. The medium was collected and secreted GOLPH2 was analyzed by sandwich ELISA. Data are normalized to control plasmids. Bars show mean and error bars show 95% CI of mean. *n* = 6, *p<0.01. **B. Cell surface presentation of GOLPH2 truncation proteins in HeLa cells.** About 48 h after transfection, HeLa cells were collected and resuspended. The cell surface distribution of GOLPH2 truncation proteins was analyzed by flow cytometry using anti-FLAG mAb. Data are normalized to control plasmids. Bars show mean and error bars show 95% CI of mean. *n* = 6, *p<0.01. **C. Expression level of GOLPH2 truncation proteins.** About 48 h after transfection, HeLa cells were collected and subjected to SDS PAGE followed by western blot. The expression level of GOLPH2 truncation proteins was detected with Western-blot using anti-FLAG mAb. The molecular weight marker is shown on the left.

Based on the predicted secondary structure and the level of sequence conservations(Figure S1 and Figure S2), we divided GOLPH2 into five regions (Figure 1A and Table 1). To initiate the structural-functional analysis of GOLPH2, we constructed GOLPH2 truncation mutants (Figure 1B). Previous studies have demonstrated that the intracellular trafficking of GOLPH2 via endosomes is closely associated with secretion when protein expression is upregulated [10]. To determine the important structural elements determining intracellular trafficking and secretion, we first measured the secretion level of GOLPH2 truncation mutants using the quantitative enzyme-linked immunosorbent assay (ELISA) for GOLPH2 [5]. As indicated in Figure 2A, the overexpression of full-length GOLPH2-FL and GOLPH2-ΔV resulted in a significantly increased secretion. However, compared with GOLPH2-FL and GOLPH2-ΔV, a significantly decreased secretion was observed in all mutants with region I deletions (GOLPH2-ΔI, GOLPH2-Δ(I-V), and GOLPH2-Δ(I-II)). These results suggested the importance of the N-terminal cytoplasmic tail and TMD of GOLPH2 in its secretion.

**Table 1:** Regions of GOLPH2.

Region No.	AA No.	Subcellular localization, predicted structure, conservation
I	1–12	Cytoplasm localized. Conserved. M1-S10 does not exist in some isoforms (UniProt # QBNBJ4).
II	13–35	Transmembrane domain. Conserved.
III	36–205	Golgi luminal. Coiled-coil. Conserved.
IV	206–348	Golgi luminal. None structured. Diversified.
V	349–401	Golgi luminal. Conserved.

Based on the sequence and structure analysis, GOLPH2 is divided into five regions. The amino acid sequence number and predicted structure is shown.

A previous study has suggested that GOLPH2 could be transiently presented to the cell surface during trafficking [10]. In the present study, we determined whether cell surface presentation was also affected by truncation. Consistent with the protein secretion changes above described, when HeLa cells were transfected with plasmids for GOLPH2-FL, GOLPH2-ΔV, and GOLPH2-Δ(IV–V), there was an increased cytoplasmic membrane presentation of the expressed proteins (Figure 2B). However, for the region I deletion mutants (GOLPH2-ΔI, GOLPH2-Δ(I–V), and GOLPH2-Δ(I–II)), no evident change was observed, even though a similar expression level of recombinant proteins was readily detected from whole cell lysates (Figure2C). Therefore, mutants with both the N-terminal cytoplasmic tail and the TMD deleted failed to transport to or localize on the cell surface.

### The TMD and cytoplasmic tail of GOLPH2 determine its Golgi localization

**Figure 3:**
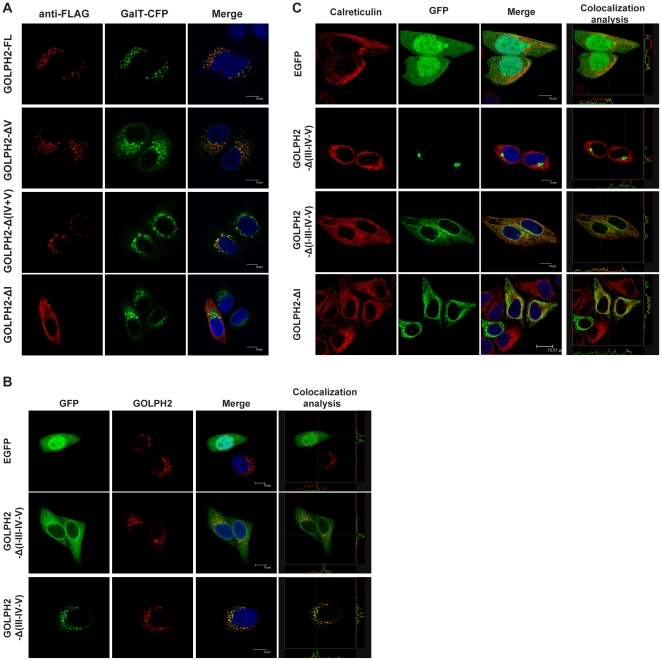
The TMD and cytoplasmic tail of GOLPH2 determine its Golgi localization. **A. Subcellular localization of GOLPH2 truncation proteins in HeLa cells.** Recombinant proteins transiently expressed in HeLa cells were stained with anti-FLAG mAb, which emits red fluorescence. Subcellular localization was imaged using a confocal microscope. β-1,4-galactosyltransferase (GalT) fused with cyan fluorescent protein was cotransfected as a Golgi localization marker (shown as a green pseudocolor). Merged images show the colocalization of GOLPH2 truncation proteins and GalT. Bars, 10 µm. **B. The TMD and cytoplasmic tail of GOLPH2 were sufficient for Golgi localization.** HeLa cells were transfected with GOLPH2-Δ(III–IV–V), GOLPH2-Δ(I–III–IV–V), and pEGFP. About 24 h after transfection, cells were treated for 4 h with cycloheximide (100 µg/mL) to inhibit protein synthesis prior to fixation and permeabilization. Subcellular localization was imaged using a confocal microscope. Endogenous GOLPH2 was probed using anti-GOLPH2 mAb as a Golgi localization marker (red fluorescence). Merged images were analyzed using the Leica confocal software. Bars, 10 µm. **C. Deletion of region I resulted in ER localization.** GOLPH2-Δ(III–IV–V), GOLPH2-Δ(I–III–IV–V), GOLPH2-ΔI, and pEGFP were transfected into HeLa cells. Subcellular localization was imaged using a confocal microscope. Calreticulin was probed as an ER marker protein (red fluorescence).

To determine the intracellular localization of these mutant proteins, plasmids encoding C-terminal FLAG-tagged GOLPH2 truncation mutants were transiently transfected into HeLa cells. The cellular localization of the recombinant proteins was then determined. *β-1,4-galactosyltransferase* (GalT) fused with cyan fluorescent protein (a gift from Dr. Jun Yuan of the Harvard Medical School, Boston, MA) at the C-terminal was used as a Golgi membrane marker [16]. As shown in Figure 3A, GOLPH2-FL, GOLPH2-ΔV, and GOLPH2-Δ(IV–V) colocalized with GalT in the Golgi. On the other hand, GOLPH2-ΔI showed dispersed localization within the cytoplasm. These results suggested that the Golgi localizing signal was located within regions I, II, and III.

To determine the location of the Golgi targeting signal for GOLPH2, two C-terminal EGFP fusion proteins were constructed. These proteins contained regions I and II of GOLPH2 (GOLPH2-Δ(III–IV–V)) and only region II (GOLPH2-Δ(I–III–IV–V)). Their cellular localizations were compared with endogenous GOLPH2 using the Leica Confocal Software. As shown in Figure 3B, GOLPH2-Δ(III–IV–V) retained Golgi localization, whereas GOLPH2-Δ(I–III–IV–V) became localized to the cytoplasm. When the localization of GOLPH2-Δ(I–III–IV–V) and GOLPH2-ΔI were compared with calreticulin (an endoplasmic reticulum (ER) marker protein), both truncated proteins were localized to the ER. These results indicated that regions I (12 amino acids) and II (23 amino acids) were sufficient to support protein Golgi localization, whereas region III is not required.

### The Golgi retention signal is in the TMD of GOLPH2, and requires a positively charged residue at the cytoplasmic end

**Figure 4:**
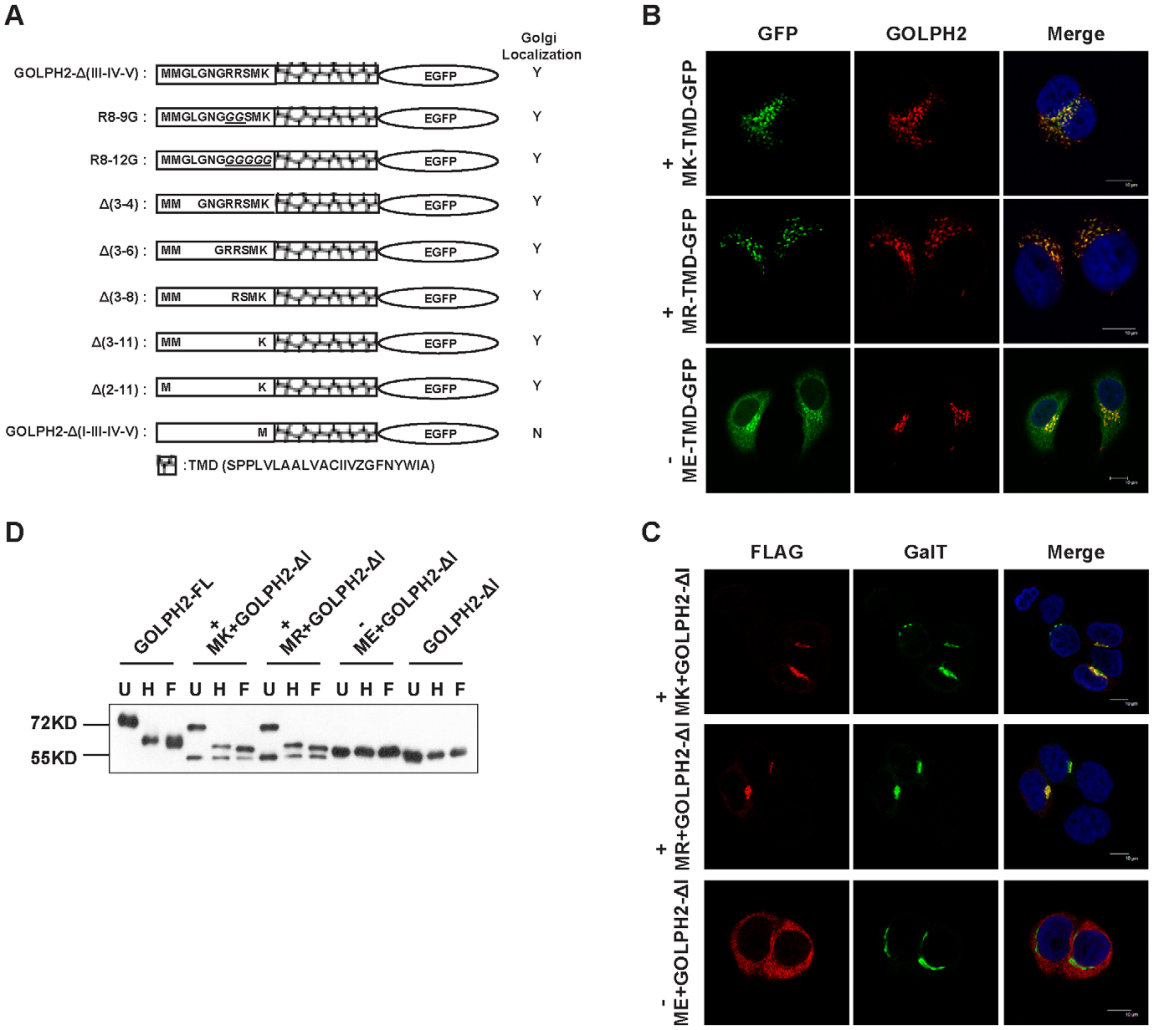
The Golgi localizing signal is in the TMD, and requires a positively charged residue. **A. Summary of fusion proteins used in the current study and their properties in cellular localization.** The amino acid sequences of mutants corresponding to the cytoplasmic domain and their cellular localization are indicated. Replacement mutants are shown above, and deletion mutants are shown below. **B. A positively charged residue at the cytoplasmic end is important for Golgi retention.** The first Lys of Δ(2–11) was replaced with positively charged Arg or negatively charged Glu. The mutant plasmids were transfected into HeLa cells, and cellular localizations were imaged using an indirect immunofluorescence microscope. Endogenous GOLPH2 was detected using anti-GOLPH2 mAb as a Golgi localization marker (red fluorescence). Merged images reveal the colocalization of endogenous GOLPH2 and mutants. Bars, 10 µm. **C. A positively charged residue at the cytoplasmic end assists Golgi localization.** Lys, Arg, and Glu were added to the N-terminal of GOLPH2-ΔI, and the subcellular localization of these mutants was imaged using a confocal microscope. GalT was used as a Golgi marker and shown as green fluorescence. **D. Endoglycosidase Digestion Assay of GOLPH2 mutants.** GOLPH2 mutants were transfected into HeLa cells, which were then harvested 48 h after transfection and lysing. The cell lysates were denatured and digested with 500 U Endo H or PNGase F for 3 h at 37°C. The molecular weight marker is shown on the left. U: untreated; H: treated with Endo H; F: treated with PNGase F.

Since the cytoplasm region of GOLPH2 (region I) is a conserved 12-amino acid polypeptide, we wondered if specific sequences in this region were required to support the Golgi localization of GOLPH2. To answer this question, we first generated two amino acid replacement mutants of the region I portion of GOLPH2-Δ(III–IV–V)-EGFP. Two arginine (Arg) residues at the 8–9 positions were replaced with two glycines (Gly) (Figure 4A, R8-9G), or five residues for the 8–12 positions (RRSMK) were replaced by five Gly (Figure 4A, R8-12G). The subcellular localization of these mutants was analyzed (Figure S3) and is summarized in Figure 4A. The results showed that the two replacement mutants retained Golgi localization, indicating that sequence specificity of region I was not required. To determine the minimum requirement of region I to support Golgi localization, a group of deletion mutants was generated. As summarized in Figure 4A (and Figure S3), two amino acids in region I, methionine-lysine (Lys), together with the TMD, were sufficient to support Golgi localization (Figure 4A, Δ(2–11)), whereas methionine alone did not (Figure 4A, GOLPH2-Δ(I–III–IV–V)).

Lys is a positively charged residue. To determine whether the nature of its charge at this position is important for Golgi localization, the first Lys followed by the TMD of GOLPH2-Δ(2–11) was replaced with another positively charged Arg or the negatively charged glutamic acid (Glu). As shown in Figure 4B, the protein with the Lys→Arg replacement retained Golgi localization (Figure 4B, MR-TMD-EGFP), whereas the Lys→Glu replacement lost Golgi localization (Figure 4B, ME-TMD-EGFP). These results suggested that a positively charged residue followed by the TMD of GOLPH2 was sufficient for Golgi localization.

GOLPH2 is glycosylated and contains N-linked carbohydrates (Figure1A). The proper carbohydrate modification of a protein depends on its correct intracellular localization. Consequently, we next verified the effect of the positively charged residue at the cytoplasmic end by comparing the status of their N-linked carbohydrate modification. The amino acids Lys, Arg, or Glu were added at the N-terminal of GOLPH2-ΔI, and the subcellular localization of these mutants was determined. Consistent with the above observations, proteins with positively charged residues at the cytoplasmic end localized to Golgi (MK+GOLPH2−ΔI MR+GOLPH2−ΔI, Figure 4C), whereas the GOLPH2 mutant with a negatively charged N-terminal residue did not (ME+GOLPH2−ΔI, Figure 4C). After treatment with Endo H and PNGase F, there was no noticeable difference in GOLPH2−ΔI and ME+GOLPH2−ΔI. This result suggested that GOLPH2−ΔI and ME+GOLPH2−ΔI were not modified by N-linked glycosylation, even though GOLPH2−ΔI was localized in the ER (Figure 4D). However, similar with GOLPH2-FL, there was a distinct decrease in molecular weight in MK+GOLPH2−ΔI and MR+GOLPH2−ΔI after digestion with Endo H and PNGase F. This finding indicated that both MK+GOLPH2−ΔI and MR+GOLPH2−ΔI were modified by N-linked glycosylation.

Therefore, the Golgi retention signal is in the TMD of GOLPH2, and requires a positively charged residue at the N-terminal cytoplasmic end.

### The TMD length is a determinant for GOLPH2 Golgi localization

**Figure 5:**
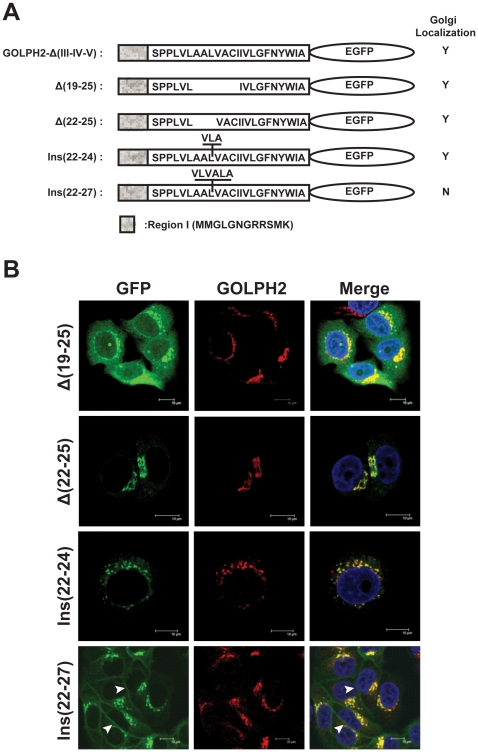
The length of hydrophobic amino acids in the TMD is important for GOLPH2 Golgi retention. **A. Mutations used in the present study.** Hydrophobic residues were deleted from or added to the TMD of GOLPH2-Δ(III–IV–V), and the amino acid sequences of the mutants are indicated. **B. Cellular localizations of fusion proteins.** The mutant plasmids were transfected into HeLa cells, and cellular localizations were viewed using a confocal microscope. Endogenous GOLPH2 was probed by anti-GOLPH2 mAb as a Golgi localization marker (red fluorescence). Merged images show the colocalization of endogenous GOLPH2 and mutants. Bars, 10 µm.

Previous studies have revealed that the length of a TMD is important for its membrane localization. Shorter TMDs of Golgi proteins prevent them from entering cholesterol-rich transport vesicles for fusion with the plasma membrane [11], [17], [18], [19]. The TMD for GOLPH2 is 23 aa long. To explore whether the length of the TMD for GOLPH2 also influences its intracellular membrane localization, the hydrophobic residues were shortened (Δ(19–25) and Δ(22–25)) or elongated (Ins(22–24) and Ins(22–27)) in the TMD (Figure 5A). The subcellular localization of these mutants was then viewed under confocal microscopy. As shown in Figure 5B, adding three hydrophobic residues (Ins(22–24), 26 aa long) and deleting four hydrophobic residues (Δ(22–25), 19 aa long) did not change the Golgi localization of GOLPH2. However, when seven hydrophobic residues were deleted (Δ(19–25), 16 aa long), the protein dispersed throughout the entire cell. When six hydrophobic residues were added (Ins(22–27), 29 aa long), some of the proteins localized to the plasma membrane (Figure 5B, Ins(22–27)). These results suggested that a 19–26 aa long TMD was sufficient to maintain Golgi localization, whereas a longer TMD facilitated transport to the plasma membrane.

### GOLPH2 exists as a dimmer

**Figure 6:**
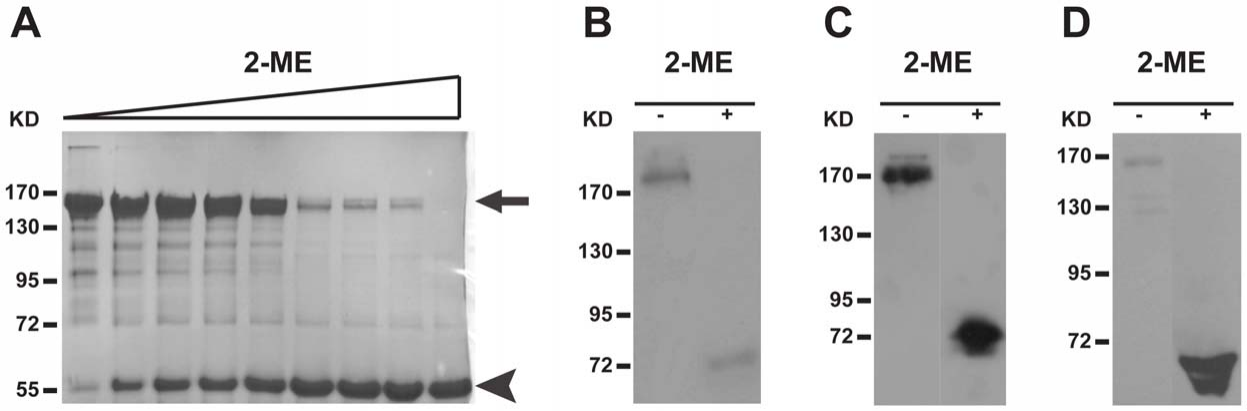
GOLPH2 forms disulfide bond-linked oligomers. **A,** GOLPH2-Δ(1–55) was purified by Ni-NTA affinity chromatography, and then boiled to a final concentration of 0 mM to 100 mM 2-mercaptoethanol. Separation by 10% SDS-PAGE followed. GOLPH2 oligomers dissociated with increased 2-mercaptoethanol. **B,** The HeLa cell lysates were boiled in the presence of 0 mM or 140 mM 2-mercaptoethanol. Samples were separated by 8% SDS-PAGE, and analyzed by immunoblotting with GOLPH2 antibody. **C** and **D**, Secreted GOLPH2 from normal human urine (C) and HeLa cell supernatant (D) were prepared under non-reducing and reducing conditions (140 mM 2-mercaptoethanol). Immunoblotting revealed oligomer formation under non-reducing conditions.

The Golgi localization of many Golgi proteins is maintained by oligomerization [14], [15]. To determine the oligomeric state of GOLPH2, purified GOLPH2-Δ(1–55) was analyzed by sodium dodecyl sulfate (SDS)-polyacrylamide gel electrophoresis (PAGE). As shown in Figure 6A, GOLPH2-Δ(1–55) migrated to higher bands at around 150 kDa under non-reducing conditions, but was concentrated mainly to the lower 55 kDa band under reducing conditions. Similarly, intracellular GOLPH2 from HeLa cell lysate (Figure 6B), secreted GOLPH2 in human urine (Figure 6C) and HeLa culture supernatant (Figure 6D) was also analyzed by SDS-PAGE and immunoblotting. In each case, the apparent molecular weight under non-reducing conditions was 2–3-fold greater than under reducing conditions. The addition of the reducing agent 2-mercaptoethanol dramatically altered the electrophoretic pattern by promoting the extensive dissociation of oligomers. These results indicated that both the endogenous and secreted forms of GOLPH2 existed as oligomers under non-reducing conditions.

**Figure 7:**
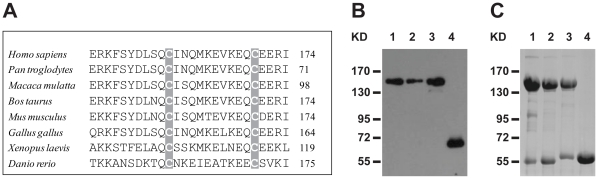
GOLPH2 forms a dimer. A, A partial amino acid sequence of the coiled-coil domain of human GOLPH2 was aligned with the sequences of the GOLPH2 proteins of different species: *Pan troglodytes* (XP_520104), *Macaca mulatta* (XP_001105085), *Bos Taurus* (XP_595216), *Mus musculus* (NP_001030294), *Gallus gallus* (XP_425035), *Xenopus laevis* (NP_001087874), and *Danio rerio* (NP_956573). The two cysteines are shaded gray. **B,** Wild-type GOLPH2 and the mutated forms were analyzed by SDS-PAGE: wild-type GOLPH2 (Line 1), single mutant C159A (Line 2), C170A (Line 3), and double mutant C159A-C170A (Line 4). HeLa cells were transfected with pCR3.1-GOLPH2-FLAG or the mutant forms. About 36 h post-transfection, the cell lysates were analyzed by SDS-PAGE under non-reducing conditions. The bands were detected by immunoblotting with FLAG antibody. **C,** Wild-type GOLPH2-Δ(1–55) and the mutants were affinity purified and resolved by SDS-PAGE under non-reduced conditions.

GOLPH2 is rich in acidic residues, and has a calculated isoelectric point of 4.9. Hence, migration is slower in Laemmli-SDS-PAGE because of the weaker binding to SDS [20]. Consequently, whether GOLPH2 oligomer is a dimer or a trimer could not be determined by the apparent molecular weight. Analysis of the protein sequences of GOLPH2 homologs revealed two consensus cysteines within the end of the coiled-coil stem (Figure 7A). To determine the composition of the GOLPH2 complex (dimer or trimer), the contributions of the two cysteines (C159 and C170) in the formation of GOLPH2 oligomer were analyzed. Cysteines were replaced with alanines individually (C159A or C170A) or together (C159A and C170A). The GOLPH2 mutants with C-terminal FLAG-tags were transfected into HeLa cells, and the expressed proteins were resolved by SDS-PAGE under non-reducing conditions, followed by immunoblotting. The single mutants (C159A or C170A) migrated to the same position of high molecular weight as wild-type GOLPH2 (Figure 7B). However, the double mutant (C159A and C170A) appeared as a monomer (Figure 7B). Similarly, purified GOLPH2-Δ(1–55) and the mutated forms expressed in *Escherichia coli* showed the same migration pattern (Figure 7C). These results suggested that both cysteines were involved in the formation of disulfide bonds in the complex, and that the arrangements of the disulfide bonds were C159-C159 and C170-C170. Such disulfide bond conformation was only possible when GOLPH2 existed as a dimer.

### The coiled-coil domain is sufficient to mediate GOLPH2 dimerization

**Figure 8:**
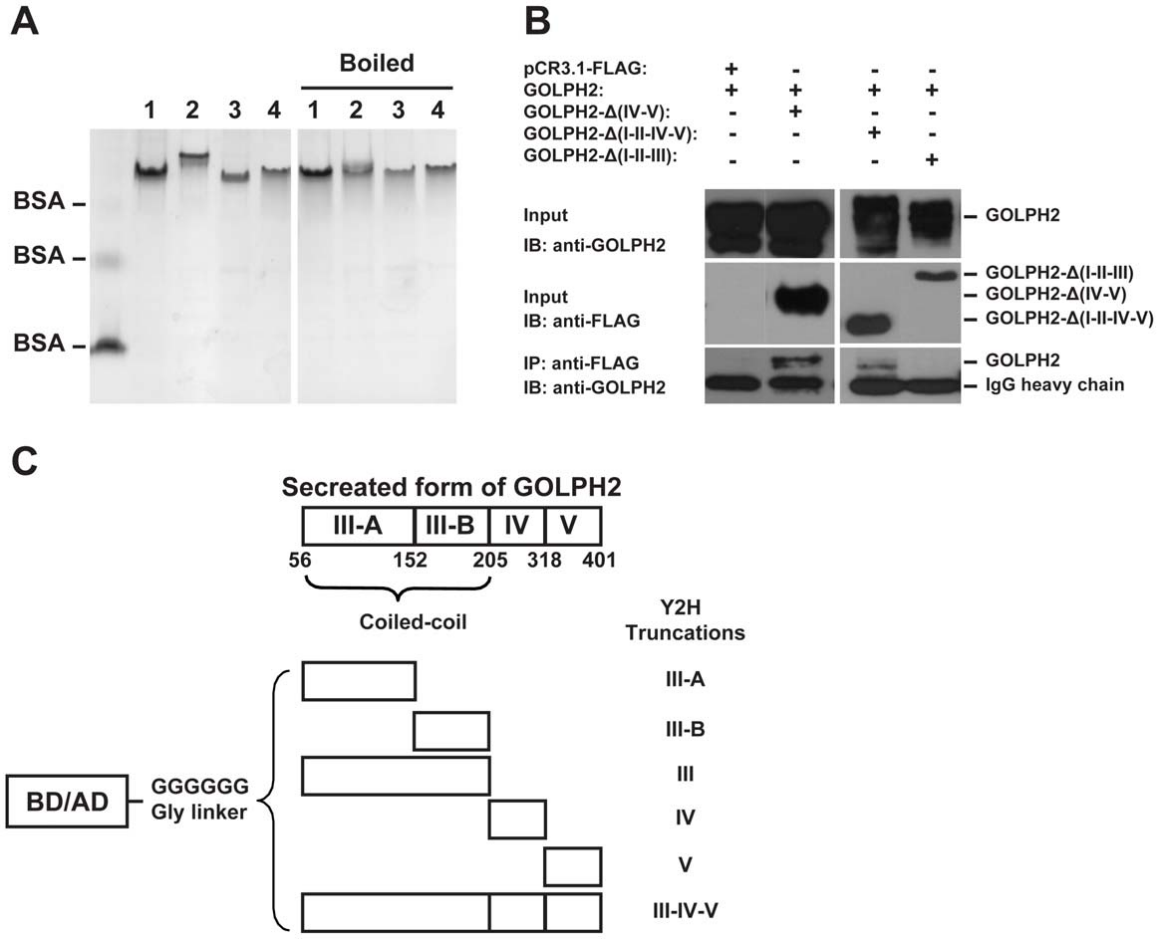
The coiled-coil domain is sufficient to mediate the formation of GOLPH2 dimer. **A,** The purified-wild type GOLPH2 (Line 1), single mutants C159A (Line 2), C170A (Line 3), and double mutants C159A–C170A (Line 4) were analyzed by native-PAGE. Samples were loaded directly on the gel or boiled with additional 2% SDS and 140 mM 2-mercaptoethanol. The BSA monomer, dimer, and tetramer forms were used as markers. **B,** The plasmids encoding GOLPH2-Δ(IV–V) (amino acids 1–205), GOLPH2–Δ(I–II–IV–V) (amino acids 56–205), and GOLPH2–Δ(I–II–III) (amino acids 206–401) were co-transfected with pCR3.1-GOLPH2-FL and expressed in HeLa cells (input). Cell lysates were captured with anti-FLAG antibody, and precipitated by Protein G Plus/Protein A agarose. The immunoprecipitates were analyzed by immunoblotting with GOLPH2 antibody. **C.** The coiled-coil domain of GOLPH2 was subdivided into two parts: III-A (amino acids 56–152) and III-B (amino acids 153–205). Each fragment of GOLPH2 was cloned into pGBKT7 and pGADT7. A six-glycine linker was inserted between them and the GAL4 DNA binding (or activation) domain.

To investigate the nature of GOLPH2 dimerization, purified GOLPH2-Δ(1–55) and its cysteine mutants were analyzed by native-PAGE. As shown in Figure 8A, the mutated GOLPH2-Δ(1–55) had the same migration pattern as the wild-type form, even after the proteins were denatured by boiling in SDS and 2-mercaptoethanol. This finding indicated that disulfide bonds were not necessary in the dimerization of GOLPH2.

To identify the structural determinant for the dimerization of GOLPH2, GOLPH2-Δ(IV–V) (amino acids 1–205), GOLPH2-Δ(I–II–IV–V) (amino acids 56–205), and GOLPH2-Δ(I–II–III) (amino acids 206–401) were co-transfected in HeLa cells with full length GOLPH2 (without FLAG tag) respectively. Co-immunoprecipitation assay using an anti-FLAG antibody followed. GOLPH2 co-precipitated with GOLPH2-Δ(IV–V) and GOLPH2-Δ(I–II–IV–V), but neither reaction was observed with GOLPH2-Δ(I–II–III) nor control plasmid pCR3.1-FLAG (Figure 8A). These results indicated that the coiled-coil domain (region III) is responsible for GOLPH2 dimerization.

A yeast two-hybrid assay was used to fine-map the regions required for GOLPH2 dimerization. As shown in Figure 8B, the coiled-coil domain (region III) of GOLPH2 was subdivided into III-A (amino acids 56–152 containing many hydrophobic residues) and III-B (amino acids 153–205 containing the two cysteines for the disulfide bonds). Truncated GOLPH2 was subcloned into both pGBKT7 and pGADT7 to create a Gal4 DNA binding domain and activation domain fusion proteins, respectively. These constructs were not self-activating and could be stably expressed in the yeast (data not shown).

As presented in Table 2, each Gal4 fusion protein was tested for its interaction with the activation domain fusion proteins. Strong interactions (++) were indicated when colonies formed on SD/-Trp/-Leu/-His/-Ade plates. Weak interactions (+) were indicated when colonies only formed on SD/-Trp/-Leu/-Ade plates supplemented with 1 mM 3-AT at 30°C for 3 d to 8 d. As predicted, the secreted form of GOLPH2-Δ(1–55) interacted with itself in the yeast. Interestingly, GOLPH2-IIIA also interacted with itself in addition to GOLPH2-III–IV–V, indicating that GOLPH2-IIIA was capable of dimerization. However, the GOLPH2-IIIB did not interact with any other truncated form of GOLPH2. These results confirmed that the coiled-coil domain was sufficient to mediate GOLPH2 dimerization, and that the interaction region was located between amino acids 56–152.

**Table 2:** Mapping the binding region of GOLPH2.

		pGADT7-GOLPH2					
		III-A	III-B	III	IV	V	III-IV-V
**pGBKT7-GOLPH2**	**III-A**	**+ +**					**+ +**
	**III-B**						
	**III**						
	**IV**						
	**V**						
	**III-IV-V**	**+ +**					**+ +**

Each of the gal4 binding domain fusion proteins of truncated GOLPH2 were tested for their interaction with the activation domain fusion proteins to truncated GOLPH2. Strong interactions (++) are indicated by growth on SD/-Trp/-Leu/-His/-Ade plates, while weak interactions (+) are indicated by growth on SD/-Trp/-Leu/-Ade plates with 1 mM 3-amino-124-triazole (3-AT).

## Discussion

Two mechanisms have been proposed for Golgi localization, namely, the bilayer-thickness and oligomerization models [14], [15]. In the present study, we demonstrated that both endogenous GOLPH2 and the secreted GOLPH2 (sGOLPH2) exist as a disulfide-bonded dimer. We then demonstrated that the coiled-coil domain of GOLPH2 was sufficient to mediate the dimerization of GOLPH2. We also provided evidence that GOLPH2 dimerization was not required for Golgi localization. Instead, the GOLPH2 Golgi localizing signals were the TMD and N-terminal cytoplasmic domain.

As a type II Golgi resident glycoprotein, GOLPH2 shares many structural features with Golgi glycosyltransferase, including a short N-terminal cytoplasmic domain, a single-pass TMD, and a relatively longer C-terminal Golgi luminal region. The Golgi targeting mechanism has been studied more extensively in Golgi glycosyltransferases than in type II glycoproteins. These studies have shown that the TMD has a major effect on Golgi localization [11], [12], [13], [14], [15], [21]. However, given that many of these enzymes exist in protein complexes, the actual targeting signals may probably reside in other members of the complex, rather than in the enzyme. Our results indicated that a fusion protein containing the GOLPH2 cytoplasmic and transmembrane domains fused with EGFP was Golgi localized. This finding suggested that regions I and II contained Golgi localizing signals, in contrast to the conclusion of Puri *et al*. They suggested that the coiled-coil domain was the Golgi-targeting signal. This suggestion was based on the observation that a fusion protein with the cytoplasmic tail and the TMD of DPPIV and the GOLPH2 coiled-coil domain remained Golgi localized [22]. This discrepancy may be explained by our second observation, showing that GOLPH2 was a dimer via coiled-coil domain interactions. Hence, it is possible that the recombinant protein generated by Puri *et al*. was localized to the Golgi through oligomerization with endogenous GOLPH2.

In analyzing the region I mutants, a number of interesting features of the cytoplasmic tail were revealed. First, most of the cytoplasmic tail was dispensable for GOLPH2 Golgi localization. The polypeptide M^1^-S^10^ does not exist in the isoform-2 of GOLPH2 (UniProt # QBNBJ4, M^11^ is used as the starting cordon). Hence, our results provided direct evidence that both isoforms 1 and 2 of GOLPH2 were capable of Golgi membrane localization. Second, earlier observations have signified that the TMD flanking residues at the cytoplasmic side are usually positively charged to assist the orientation of the protein as well as the steady interaction between the TMD and the negatively charged proteoglycan in the Golgi membrane. Consistent with this finding, we found that GOLPH2 isoform-2, which contains only two residues (MK) in its cytoplasmic side, required at least one positively charged residue. Replacing Lys with the negatively charged Glu diminished its Golgi localizing capacity. However, this was not necessary for GOLPH2 isoform-1, because replacing R^8^RSMK^12^ with G^8^GGGG^12^ (Figure 4A, R8-9G) caused it to remain Golgi localized.

The TMD as a Golgi retention signal has first been found in a-2,6-sialyltransferase [13], [23]. Subsequently, the bilayer thickness model is introduced to explain Golgi localization [14], [21], [24]. This model is based on the observations that Golgi proteins generally have shorter TMDs than plasma membrane proteins. On the other hand, the lipid bilayer increases in thickness from the ER through the Golgi to the plasma membrane due to increasing concentrations of cholesterol and sphingolipids. The cholesterol also changes the bilayer properties, which augment the energetic penalty for incorporating short TMDs into cholesterol-enriched domains [24]. As a result, TMD lengthening leads to increased cell surface expression. Evidence presented in the present study supported this theory. We found that shortening the GOLPH2 TMD to 19 aa or elongating it to 26 aa did not affect its Golgi localization. However, when the length was increased to 29 aa, the protein became partially localized to the plasma membrane.

Bachert *et al*. have reported that upregulated intracellular GOLPH2 expression enhanced its intracellular trafficking, most likely via an endosomal pathway[10]. This phenomenon resulted in the secretion of a cleaved truncated GOLPH2. In the present study, GOLPH2-ΔI, GOLPH2-Δ(I–II), and GOLPH2-Δ(I–V), which lost Golgi localization, failed to traffic and secrete normally as GOLPH2-FL and GOLPH2-ΔV did. This evidence suggested that the intracellular trafficking and secretion of GOLPH2 was closely related to its Golgi localization.

Another interesting observation came from GOLPH2-Δ(I–III–IV–V), a protein with its cytoplasmic tail completely removed. In transiently transfected cells, some of the proteins were seemingly nuclear membrane localized (Figure 3B, GOLPH2-Δ(I–III–IV–V)). This observation was not found in the other cytoplasmic tail-deleted mutant (Figure 3A, GOLPH2-ΔI). The difference may be attributed to the domain following the TMD, but further investigation is needed.

The two cysteines C159 and C170 (human GOLPH2) are conserved among the orthologs of GOLPH2. Hence, the observed formation of inter-protein disulfide bonds between these cysteines in the present study was intriguing; these covalent linkages were not required for GOLPH2 dimer formation. These findings suggested that the disulfide bonds only served to provide covalent links to stabilize the dimer, whereas hydrophobic interactions between the coiled-coil domains are the major forces in protein-protein interactions. Indeed, our Y2H experiments indicated that GOLPH2-IIIB, the portion that did not contain cysteines, was capable of mediating GOLPH2 complex formation. We have recently identified clusterin as one of the proteins interacting with GOLPH2 through the coiled-coil domain of GOLPH2 [25]. In the current study, we have also observed coiled-coil domain-dependent higher-order oligomerized GOLPH2 using dynamic light scattering and immunoblotting experiments. These observations further suggested that the highly conserved coiled-coil domain of GOLPH2 was a major structure determinant of the protein. The biological importance of these structural features, including the highly conserved two cysteines, is yet to be discovered.

**Figure 9:**
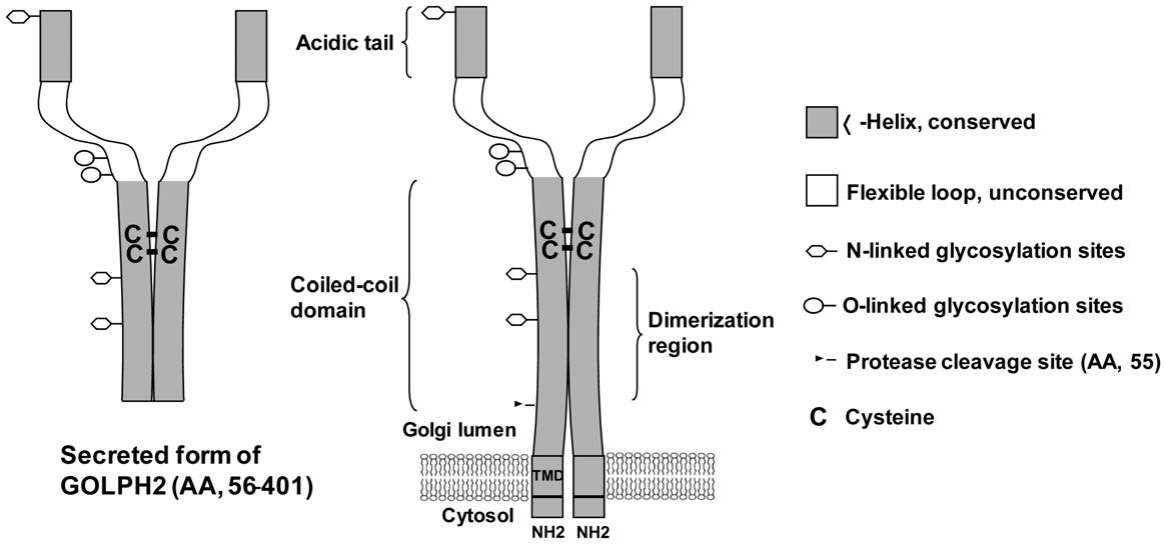
A proposed model of a GOLPH2 dimer. We speculate that both endogenous and secreted GOLPH2 are dimerized through the center of the coiled-coil domain (amino acids 56–152). The monomers are linked by disulfide bonds (C^159^-C^159^ and C^170^-C^170^). The gray area shows the conserved sequence. Five potential locations of N- and O-linked glycosylation sites are also shown.

In summary, our study indicated that the TMD is the structure determinant for GOLPH2 Golgi localization and trafficking, and that the coiled-coil domain of GOLPH2 may assist Golgi localization via oligomerization. We thereby propose a structure model of GOLPH2 (Figure 9). Based on this model, regardless of the biological function that GOLPH2 may perform, GOLPH2 functions as a dimer. Such structural characteristics should be taken into account when analyzing interactions between GOLPH2 and its interacting partners, such as clusterin [25]. Interestingly, the sequences in regions I, II, and III are also highly conserved. This finding suggests that besides being responsible for intracellular localization and dimer formation, these regions may also be important for the biological function. Nevertheless, although more structural details of GOLPH2 remain to be clarified (including the protein crystal structure), the current model indicates that the secreted GOLPH2, a potential hepatocellular cancer serum marker, is a dimer. This knowledge could be valuable in the development of GOLPH2-based diagnostic methods, and in future structure-function studies.

## Materials and Methods

### Cell Culture

HeLa cell (ATCC, CCL-2) monolayers were maintained in the exponential growth phase in Dulbecco's modified Eagle's medium (Gibco, USA) supplemented with 10% fetal bovine serum (Hyclone, USA), 100 units/mL penicillin, and 0.1% (w/v) streptomycin.

### Plasmid Construction, Mutagenesis, and Protein Expression

The full-length cDNA of human GOLPH2 was generated by RT-PCR from the total RNA of Huh7 cells (a gift from Dr. Wong-Staal, UC-San Diego). Based on the predicted secondary structure and the level of sequence conservation, we divided GOLPH2 into five regions. As shown in Figure 1B, different GOLPH2 regions were amplified by PCR and cloned into the pCR3.1 vector (Invitrogen,USA) with an in-frame FLAG tag encoded at the C-terminus. A BamH I site was introduced into GOLPH2-Δ(IV–V) and GOLPH2-ΔI according to the Quickchange protocols (Stratagene, USA) using the forward primer 5̀-GAGCTCCCGGAGCGTGGATCCCTCCAGACACGGATCAT-3̀ (reverse compliment primers are not shown). The EGFP coding region was cloned into GOLPH2-Δ(IV–V) and GOLPH2-ΔI in site with BamH I and EcoR I to generate GOLPH2-Δ(III–IV–V) and GOLPH2-Δ(I–III–IV–V) mutants. Mutants based on GOLPH2-Δ(III–IV–V) and GOLPH2-Δ(I–III–IV–V) were constructed based on the manufacturer's instructions (Stratagene, USA). The cysteines at positions 159 and 170 in the coiled-coil domain of GOLPH2 were mutated to alanines using a QuickChange site-directed mutagenesis kit (Stratagene). Three different mutants were created for GOLPH2 in pCR3.1-uni and GOLPH2-Δ(1–55) in pET22b (+), including C159A, C170A, and C159A-C170A. All constructs were verified by sequencing. The GOLPH2-Δ(1–55) fusion protein and mutated forms (C159A, C170A, and C159A-C170A) were expressed in *E. coil* BL21 (DE3), and purified by Ni-NTA affinity chromatography (Qiagen) according to the manufacturer's instructions.

### Flow Cytometry and Confocal Microscopy

For flow cytometry analysis, cells were harvested using 0.25% trypsin with EDTA (Gibco, USA) 48 h after transfection. The cells were thrice washed with cold phosphate-buffered saline (PBS). Cell suspensions (1×10^6^ cells/mL) were incubated with anti-FLAG monoclonal antibody M2 (Sigma, USA) for 30 min on ice, followed by three washes in PBS. The cells were then incubated for 30 min on ice with fluorescein isothiocyanate-conjugated sheep anti-mouse immunoglobulin (Chemicon, USA), and analyzed using a FACSCalibur analog analyzer (Becton Dickinson) by the standard protocol.

For confocal microscopy, cells were seeded onto cover slides 24 h after transfection. After overnight culture, some slides were directly fixed in 4% paraformaldehyde in PBS for 15 min, followed by permeabilization with 0.5% Triton X-100. These cells were then stained in diluted antibodies. Other slides were first treated for 4 h in cycloheximide (100 µg/mL) to inhibit protein synthesis prior to fixation, followed by antibody staining. The FLAG epitope in the fusion proteins was detected using anti-FLAG monoclonal antibody M2 (Sigma, USA), followed by sheep anti-mouse immunoglobulin conjugated to fluorescein isothiocyanate (Chemicon, USA). Endogenous GOLPH2 was detected using an anti-GOLPH2 monoclonal antibody, as previously described [5], and a sheep anti-mouse immunoglobulin conjugated to rhodamine (Chemicon, USA). The cells were then incubated for 5 min with 4′,-6-diamidino-2-phenylindole (KPL, USA) and viewed using a confocal microscope (TCS SP2 AOBS, Leica, Switzerland).

### Immunoprecipitation

HeLa cells were transfected with expression vectors. After 36 h, extracts were prepared by collecting a confluent 6-well plate of HeLa cells into 150 µL of 1% Triton-X100 lysis buffer (pH 7.3 20 mM HEPES, 100 mM KCl, 5 mM EDTA, and 1% Triton X-100). The cells were incubated for 20 min at 4°C with gentle agitation. The lysates were centrifuged at 13 000×*g* for 20 min at 4°C. Anti-FLAG antibody M2 (2 µg, Sigma) was added to the supernatant, which was incubated overnight at 4°C. About 30 µL of a 50% suspension of protein A/G beads (Calbiochem) was added to the supernatant, which was incubated for 2 h at 4°C. The beads were washed five times with the wash buffer (pH 7.3 20 mM HEPES, 100 mM KCl, 5 mM EDTA, and 0.2% Triton X-100). Finally, the beads were resuspended in 50 µL of PBS and boiled with 12 µL of 5X Laemmli sample buffer for 10 min. The supernatant (25 µL per lane) was analyzed by SDS-PAGE, and the separated protein bands were transferred onto a polyvinylidene fluoride membrane (Millipore). The membrane was blocked for 1 h in PBS with 0.05% Tween-20 containing 5% milk, and then incubated with the antibody as needed. The bound antibodies were detected with horseradish peroxidase-conjugated rabbit anti-mouse IgG and enhanced chemiluminescence (Millipore).

### Endoglycosidase Digestion Assay

Truncation mutants were transiently transfected into HeLa cells with Lipofectamine 2000 (Invitrogen) following the manufacturer's instructions. About 48 h after transfection, the HeLa cells were collected and lysed. The cell lysates were denatured in denaturing buffer (5% SDS and 10% 2-mercaptoethnaol) for 5 min at 100°C, cooled, and then mixed with Endo H reaction buffer (pH 5.5 0.5 M sodium citrate) or PNGase F reaction buffer (pH 7.5 0.5 M sodium phosphate and 10% NP-40) at 1 part reaction buffer to 10 parts lysate. The samples were then digested with 500 U Endo H (NEB, USA) or PNGase F (NEB, USA) for 3 h at 37°C. The reaction was stopped by adding the sample buffer containing 2-mercaptoethanol, and the mixture was again incubated at 100°C for 5 min. Western blot analysis was performed to detect the digested samples with anti-FLAG mAb (Sigma, USA).

### GOLPH2 secretion assay

HeLa cells were transfected with GOLPH2 mutation expression plasmids. About 24 h post-transfection, the cells were thrice washed with PBS and cultured in fresh medium. The medium was collected 24 h later and briefly centrifuged to remove contaminating cells. Secreted GOLPH2 was analyzed using a sandwich ELISA as previously described [5]. In brief, ELISA plates (CoStar, USA) were precoated with mouse anti-GOLPH2 mAb and blocked with 2% (w/v) bovine serum albumin. The samples were incubated for 2 h at room temperature, and were detected by rabbit anti-GOLPH2 polyclonal antibody, followed by horseradish peroxidase-conjugated goat anti-rabbit IgG (Pierce, USA). Absorbance was measured at 450 nm using a microplate reader (Bio-Tek, USA).

### Liquid chromatography-tandem mass spectrometry (MS/MS)

Full-length GOLPH2-FLAG was immunoprecipitated from HeLa cells transfected with the vector expressing GOLPH2-FLAG. The mixture of proteins was resolved by SDS-PAGE under non-reducing conditions, and visualized by Coomassie blue staining. The GOLPH2-FLAG oligomer band was excised, in-gel digested with trypsin, and analyzed by liquid chromatography-MS/MS on a ProteomeX-LTQ mass spectrometer (Thermo Fisher Scientific) according to previously described methods [26], [27] The peptide sequences were identified by a data-dependent MS/MS model using a SEQUEST algorithm.

### Yeast two-hybrid

A yeast two-hybrid (Y2H) assay for protein-protein interactions was performed following the manufacturer's instructions (Matchmaker™ GAL4 Two-Hybrid System 3, Clonetech). A panel of truncated mutants was subcloned from the C-terminal luminal region of GOLPH2 (amino acids 56–401) by PCR amplification. The mutants were inserted into pGBKT7 and pGADT7 with fusion to the DNA-binding domain and the activation domain, respectively. A six-Gly linker was added between truncated GOLPH2 and these domains to increase the fusion protein flexibility during interaction. Small-scale yeast mating was performed. AH109 yeast cells pretransformed with truncated GOLPH2 in pGBKT7 were mated with Y187 yeast cells pretransformed with truncated GOLPH2 in pGADT7. Mated yeast cells were spread onto minimal SD agar plates, and positive clones were screen on SD/-Trp/-Leu/-His/-Ade or SD/-Trp/-Leu/-Ade plates with 1 mM 3-amino-124-triazole (3-AT, Sigma) at 30°C for 3 d to 8 d. Mating of AH109 yeast expressing pGBKT7-53 to Y187 yeast expressing pGADT7-T was performed as a positive control. Empty vectors pGBKT7 and pGADT7 were used as negative controls.

## Supporting Information

Figure S1 **Sequence alignment of GOLPH2 gene.** Protein sequence homologenes of GOLPH2 from different organisms were aligned. The Pairwise alignment scores and protein access numbers of NCBI are shown.
(TIF)

Figure S2 **Structure prediction of GOLPH2. A,** Prediction of transmembrane helices in GOLPH2 with the TMHMM program (http://www.cbs.dtu.dk/services/TMHMM/). **B**, Prediction of coiled-coil regions in GOLPH2 with the Paircoil program (http://groups.csail.mit.edu/cb/paircoil/cgi-bin/paircoil.cgi).
(TIF)

Figure S3 **Cellular localization of fusion proteins with mutations in the cytoplasmic domain.** The mutant plasmids were transfected into HeLa cells, and cellular localizations were viewed using a confocal microscope. Endogenous GOLPH2 was probed using anti-GOLPH2 mAb as a Golgi localization marker, indicated as red fluorescence. Merged images show the colocalization of endogenous GOLPH2 and mutants. Bars, 10 um.
(TIF)
