# Supplementary figures and images for: The Golgi Localization of GOLPH2 (GP73/GOLM1) Is Determined by the Transmembrane and Cytoplamic Sequences
Source: PLoS One. 2011 Nov 29;6(11):e28207. doi: 10.1371/journal.pone.0028207 (PMC3226628; doi:10.1371/journal.pone.0028207)

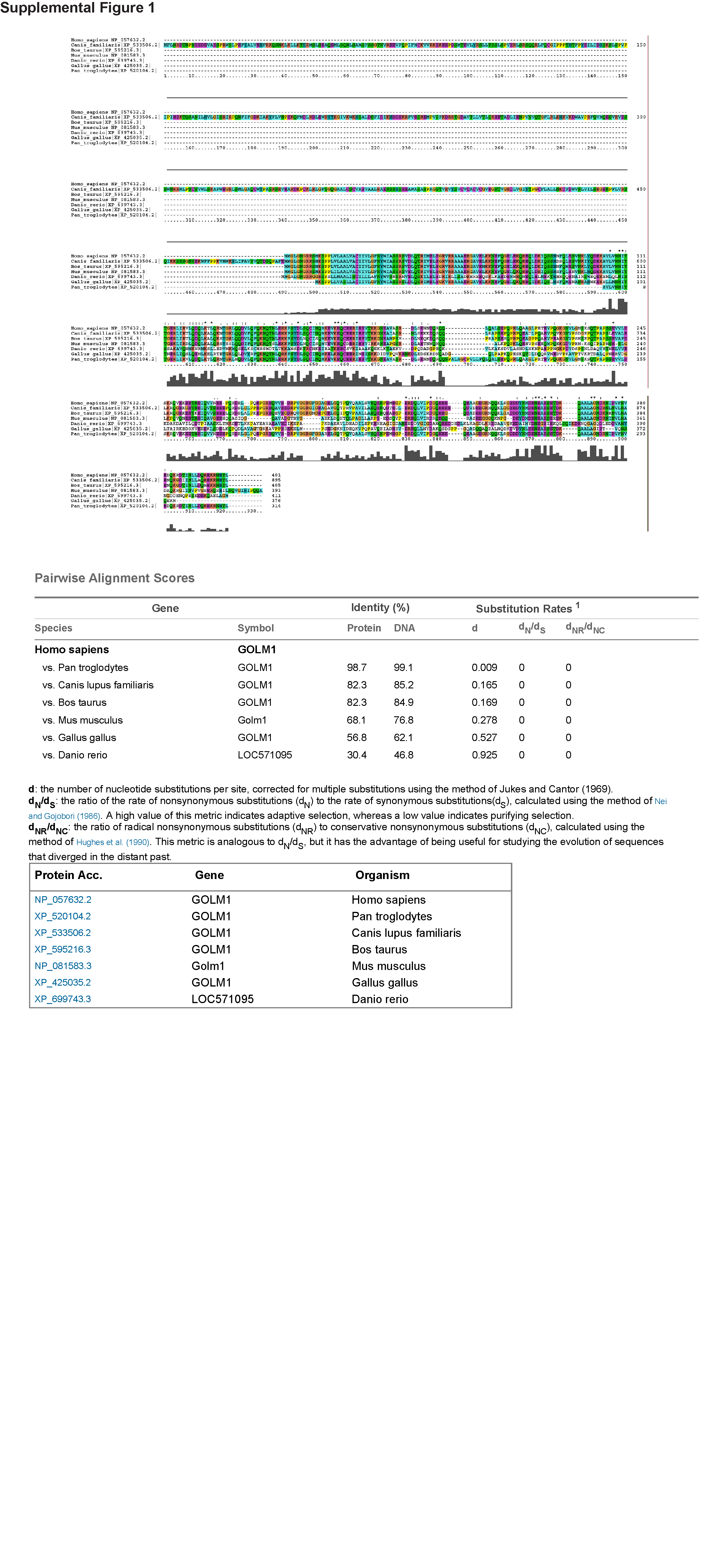

Supplement: Figure S1 — Sequence alignment of GOLPH2 gene. Protein sequence homologenes of GOLPH2 from different organisms were aligned. The Pairwise alignment scores and protein access numbers of NCBI are shown. (TIF) [file pone.0028207.s001.tif]

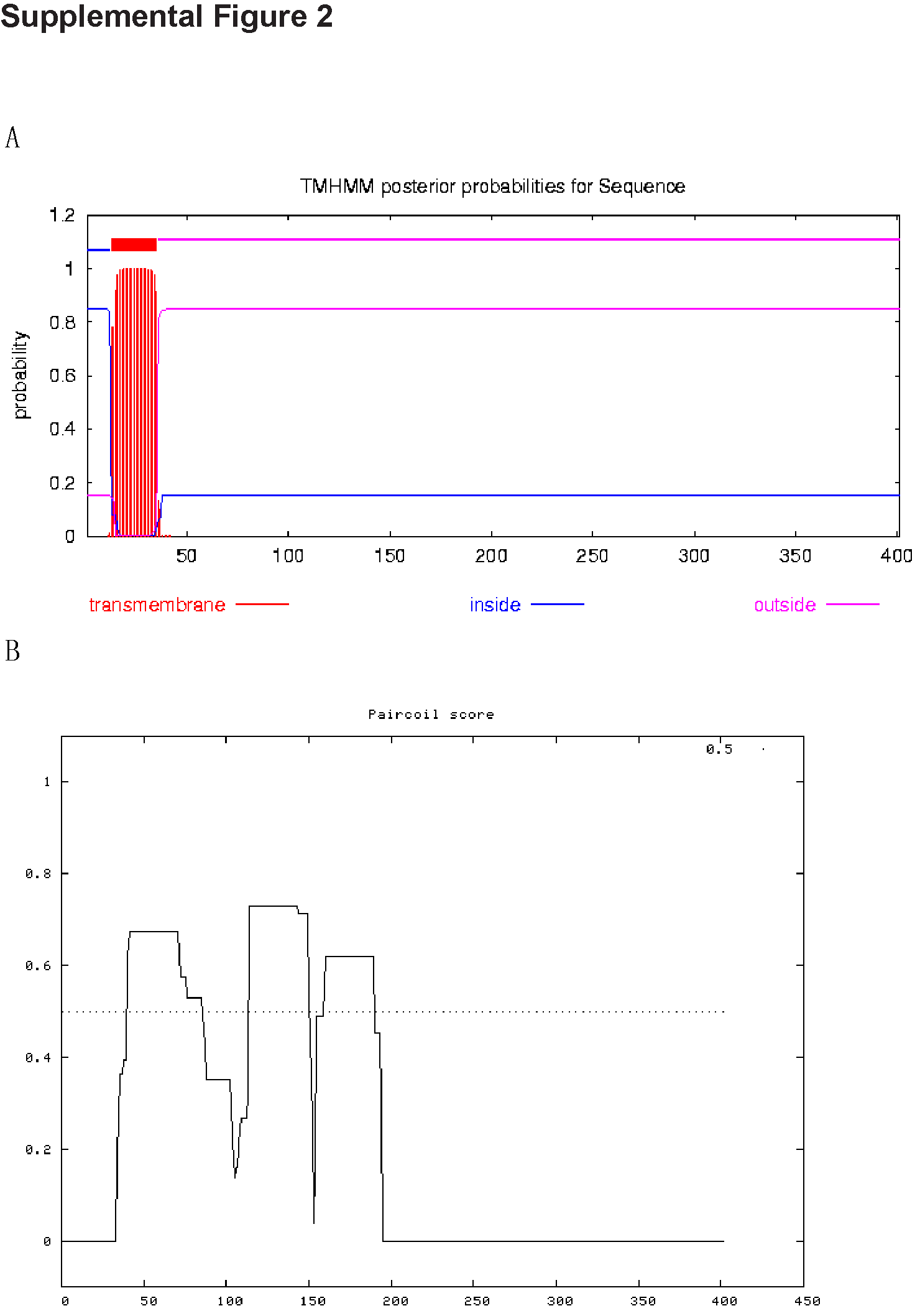

Supplement: Figure S2 — Structure prediction of GOLPH2. A, Prediction of transmembrane helices in GOLPH2 with the TMHMM program (http://www.cbs.dtu.dk/services/TMHMM/). B, Prediction of coiled-coil regions in GOLPH2 with the Paircoil program (http://groups.csail.mit.edu/cb/paircoil/cgi-bin/paircoil.cgi). (TIF) [file pone.0028207.s002.tif]

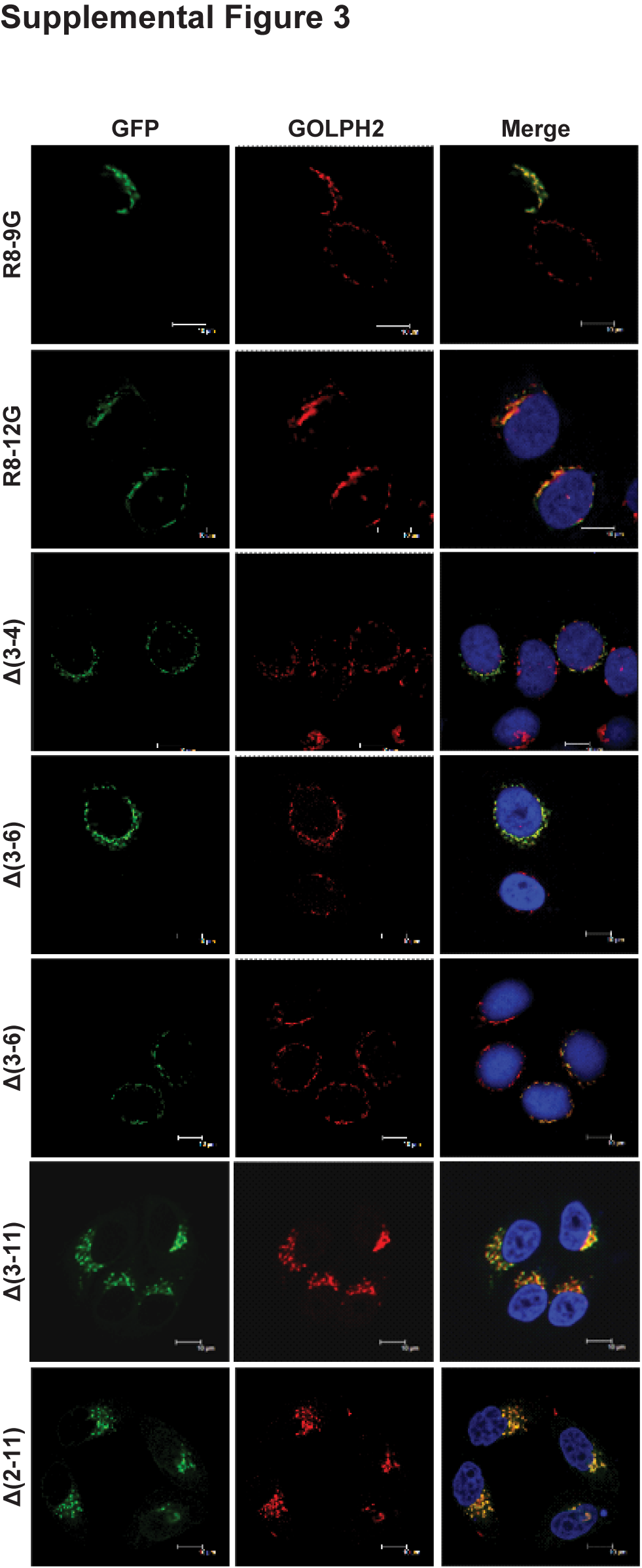

Supplement: Figure S3 — Cellular localization of fusion proteins with mutations in the cytoplasmic domain. The mutant plasmids were transfected into HeLa cells, and cellular localizations were viewed using a confocal microscope. Endogenous GOLPH2 was probed using anti-GOLPH2 mAb as a Golgi localization marker, indicated as red fluorescence. Merged images show the colocalization of endogenous GOLPH2 and mutants. Bars, 10 um. (TIF) [file pone.0028207.s003.tif]
